# A novel potential biomarker for metabolic syndrome in Chinese adults: Circulating protein disulfide isomerase family A, member 4

**DOI:** 10.1371/journal.pone.0179963

**Published:** 2017-06-26

**Authors:** Chu-Yen Chien, Yi-Jen Hung, Yi-Shing Shieh, Chang-Hsun Hsieh, Chieh-Hua Lu, Fu-Huang Lin, Sheng-Chiang Su, Chien-Hsing Lee

**Affiliations:** 1 Graduate Institute of Medical Sciences, National Defense Medical Center, Taipei, Taiwan, R.O.C; 2 Division of Endocrinology and Metabolism, Tri-Service General Hospital, National Defense Medical Center, Taipei, Taiwan, R.O.C; 3 School of Dentistry, National Defense Medical Center, Taipei, Taiwan, R.O.C; 4 Department of Oral Diagnosis and Pathology, Tri-Service General Hospital, Taipei, Taiwan, R.O.C; 5 School of Public Health, National Defense Medical Center, Taipei, Taiwan, R.O.C; The University of Texas at El Paso, UNITED STATES

## Abstract

**Background/Objectives:**

Protein disulfide isomerase (PDI) family members are specific endoplasmic reticulum proteins that are involved in the pathogenesis of numerous diseases including neurodegenerative diseases, cancer and obesity. However, the metabolic effects of PDIA4 remain unclear in humans. The aims of this study were to investigate the associations of serum PDIA4 with the metabolic syndrome (MetS) and its components in Chinese adults.

**Subjects/Methods:**

A total of 669 adults (399 men and 270 women) were recruited. Serum PDIA4 concentrations and biochemical variables were recorded. Insulin sensitivity and β-cell function were examined by homeostasis model assessment. MetS was defined based on the modified National Cholesterol Education Program Adult Treatment Panel III criteria for Asia Pacific.

**Results:**

The participants with MetS had significantly higher serum PDIA4 levels than those without MetS (*P<*0.001). After adjustments, the individuals with the highest PDIA4 tertile were associated with a higher risk of MetS than those with the lowest tertile (OR = 4.83, 95% CI: 2.71–8.60). The concentration of PDIA4 showed a stepwise increase with the components of MetS (P<0.001 for trend). The individuals with the highest PDIA4 tertile were significantly associated with waist circumference (OR = 2.41, 95% CI 1.34–4.32), blood pressure (OR = 2.71, 95% CI 1.57–4.67), fasting glucose concentration (OR = 3.17, 95% CI 1.80–5.57), and serum triglycerides (OR = 4.12, 95% CI 2.30–7.37) than those with the lowest tertile. At cutoff point of 15.24 ng/ml, the diagnostic sensitivity and specificity of PDIA4 for the metabolic syndrome were 67 and 72%, respectively, in male patients and 60 and 78%, respectively, in female patients. Finally, the result showed that PDIA4 had a significantly higher area under the curve compared with blood pressure to detect MetS using receiver operating characteristic analysis.

**Conclusions:**

Serum PDIA4 concentrations are closely associated to MetS and its components in Chinese adults.

## Introduction

Metabolic syndrome (MetS) is characterized by a cluster of metabolic abnormalities that includes abdominal obesity, dyslipidemia, hyperglycemia, and elevated blood pressure and associate with the risk of cardiovascular disease and type 2 diabetes mellitus.[1, 2] MetS is a major public health burden, with nearly a quarter of the world’s adult population being affected.[3] Depending on the criteria used, the prevalence of MetS in adults the United States and Europe is around 20–30%,[4, 5] and around 10–20% in Asia.[6, 7] In a recent study, Hwang et al. reported that the prevalence of MetS among Chinese adults is 20% in men and 15.3% in women. Obesity and insulin resistance have long been considered to be the most important components in the pathogenesis of MetS, and atherogenic dyslipidemia, hypertension, a proinflammatory, prothrombotic environment, and endoplasmic reticulum (ER) stress typically promoted by obesity and insulin resistance have been reported to be closely associated with MetS.[8–10] Recently, unresolved ER stress has been implicated in a variety of metabolic disorders including obesity and type 2 diabetes.[11]

The ER is a central organelle in cells responsible for protein folding and trafficking, lipid synthesis, and cellular calcium homeostasis. An increasing number of studies have shown that ER stress and unfolded protein response (UPR) signaling are associated with pathophysiological and metabolic changes, including obesity, type 2 diabetes, lipid metabolism, atherosclerosis, heart and liver diseases, neurodegenerative disorders and cancer.[12, 13] Recent evidence suggests that ER stress markers such as glucose-regulated protein GRP78 (BiP), calreticulin, calnexin, and protein disulfide isomerases (PDIs) can be activated in response to MetS in multiple organs, including the hypothalamus, liver, adipose tissue, muscle and pancreatic β cells.[13, 14] For example, ER stress in adipose tissue has been shown to inhibit insulin signaling, repress lipolysis, and alter secretion of adipokines such as insulin receptor substrate (IRS) 1 and IRS2. The chemical inhibition of ER stress has been shown to improve insulin sensitivity in obese mice.[15, 16] Although the ER stress hypothesis is supported by strong evidence in animal models, the relevance of ER stress to human MetS remains unclear.

Recent studies have shown that some groups of specific ER proteins, namely the PDI family, can restore physiological ER function by means of folding, transporting, recognition, dislocation and degradation machinery.[17, 18] Given the importance of PDIs in various cellular functions, PDI dysfunction may be related to the pathogenesis of numerous diseases including neurodegenerative diseases, cancer, thrombus formation and lipid metabolism.[19] Furthermore, the expression of PDIs in adipose tissue has been shown to be upregulated in obese subjects compared to lean subjects, indicating that the PDI family may also be involved in metabolic regulation.[20] Members of the PDI family, such as PDIA4, have been reported to be located on the cytoplasm and nucleus, and also to be secreted.[21] The ER-related protein, PDIA4, functions in mediating disulfide bond formation, and to have a break down, isomerization and chaperoning effect.[18] Although less is known about the physiological role of PDIA4, several studies have indicated that this gene is induced following ER stress.[22, 23] It is known that ER stress is upregulated during the development of MetS in multiple organs, however, whether ER stress-induced PDIA4 is involved in MetS remains poorly understood. Thus, the goal of this cross-sectional study was to assess whether serum PDIA4 levels are associated with the components of MetS in humans.

## Materials and methods

### Inclusion and exclusion criteria

This study was approved by the Internal Review Board of the Ethics Committee of the Tri-Service General Hospital, and all enrolled subjects gave written informed consent. The criteria for exclusion and inclusion were modified from our previous study [24]. Ambulatory adults were recruited from the outpatient clinics of Tri-Service General Hospital, Taipei, Taiwan. The criteria for inclusion into this study included an age from 20 years to 70 years, and a body mass index (BMI) of less than 35 kg/m^2^. The criteria for inclusion into this study also included the absence of infections within the previous 12 weeks, absence of taking oral anti-diabetic agents or any types of statins, absence of encountering stroke or myocardial infarction or angina and absence of malignant tumor history before participating in this study. The exclusion criteria included women who were receiving estrogen supplement therapy, an abnormal serum aspartate aminotransferase or alanine aminotransferase level (2.5 times above the upper normal ranges), acute or chronic pancreatitis, a history of any type of diabetes, cerebrovascular accidents, myocardial infarction or heart failure, autoimmune disorders or psychiatric diseases including mood disorders and alcoholism, taking concomitant drugs such as systemic steroids or any history of major surgery. Finally, a total of 669 adults were enrolled in this study. The height, weight, BMI, waist circumference, systolic and diastolic blood pressure of the subjects were recorded when collecting blood samples. Personal habits and past histories were also recorded. To further reduce extraneous variations between subjects, they were instructed to consume their usual dietary pattern during the 3 days before fasting blood samples were collected.

### Laboratory measurements

Laboratory measurements were performed as described in detail previously in our study [24]. Following a 10-h fast, blood samples were obtained during the 75-g oral glucose tolerance test (OGTT). Some of the samples were used for general biochemistry and some for measurements of serum total cholesterol, triglycerides (TG) and low-density lipoprotein (LDL) cholesterol using the enzymatic colorimetric method on a Roche Cobas C501 Chemistry Analyzer (Diamond Diagnostics- USA). The intra- and inter-assay coefficients of variation (CVs) for LDL cholesterol were 0.71% and 1.2%, respectively, and 0.8% and 1.7%, respectively, for total cholesterol. Serum levels of high-density lipoprotein (HDL) cholesterol were determined using an enzymatic colorimetric assay method after dextran sulfate precipitation. The intra- and inter-assay CVs for HDL cholesterol were 0.9% and 1.85%, respectively. In addition, fasting plasma glucose and insulin concentrations as well as 2-h plasma glucose and insulin levels in the OGTT were measured. Plasma glucose concentrations were determined using the hexokinase method on a Roche Cobas C501 Chemistry Analyzer (Diamond Diagnostics- USA). The intra- and inter-assay CVs for glucose were 0.9% and 1.8%, respectively. Plasma insulin was measured using a commercial immunoradiometric kit (BioSource Europe, Nivelles, Belgium), and the intra- and inter-assay CVs were 2.2% and 6.5%, respectively. Serum levels of high-sensitivity C-reactive protein (hsCRP) were measured using a Tinaquant (latex) high-sensitivity assay (Roche, Mannheim, Germany). The intra- and inter-assay CVs for hsCRP were 3.7% and 4.9%, respectively. Serum uric acid, creatinine and hemoglobin A1c (HbA1c) were measured, with HbA1c being determined using a Bio-Rad Variant II automatic analyzer (Bio-Rad Diagnostic Group, Los Angeles, CA, USA).

### Measurement of PDIA4 levels

Serum levels of PDIA4 protein were measured using a sandwich ELISA. This method was validated according to the Food and Drug Administration guidelines in a previous study (with intra- and inter-assay CVs of <10% and <12%, respectively).[25] Briefly, the microtiter plate provided in the kit was pre-coated with an antibody specific to PDIA4 (ELISA Kit for PDIA4 [USCN Life Science Inc., USA; Cat. No. SED774Hu]). One hundred μl of standards or samples were then added to the appropriate microtiter plate wells with a biotin-conjugated antibody specific to PDIA4 and incubated for 2 h at 37°C. Avidin conjugated to horseradish peroxidase was then added to each microplate well and allowed to incubated, followed by aspiration, the addition of 100 μl of Detection Reagent A, and incubation for 1 h at 37°C. After washing three times, 100 μl of Detection Reagent B was added followed by incubation for 30 min at 37°C. After washing five times, 90 μl of TMB Substrate Solution was added followed by incubation for 15–25 min at 37°C. Subsequently, only the wells that contained PDIA4, biotin-conjugated antibodies and enzyme-conjugated avidin changed color. The enzyme-substrate reaction was terminated by the addition of 50 μl sulfuric acid solution, and the color change was measured spectrophotometrically at a wavelength of 450 nm ± 10 nm. The concentration of PDIA4 in the samples was then determined by comparing the O.D. of the samples to the standard curve. Measurements were repeated at least three times. The indices of β-cell function (HOMA-2β) and hepatic insulin resistance (HOMA-2IR) was calculated using computer software (HOMA Calculator v2.2.2).[26]

### Definition of metabolic syndrome

MetS was defined based on the modified National Cholesterol Education Program Adult Treatment Panel III criteria for Asia Pacific.[27] Individuals with three or more of the following criteria were classified as having MetS: 1) overweight (BMI ≥24 kg/m^2^) and obese (BMI ≥27 kg/m^2^); 2) waist circumference of ≥90 cm in men and ≥80 cm in women; 3) TG ≥150 mg/dl; 4) HDL <40 mg/dl for men and <50 mg/dl for women; 5) systolic blood pressure ≥130 mmHg or diastolic blood pressure ≥85 mmHg or the current use of antihypertensive drugs; and (6) fasting blood glucose ≥100 mg/dl or the current use of antihyperglycemic drugs.

### Statistical analysis

All statistical analyses were performed using SPSS version 20.0 for Windows (SPSS Inc., Chicago, IL, USA). A P value of less than 0.05 was considered to indicate statistical significance. Comparisons between two groups were performed using the *t*-test. Serum PDIA4 concentrations were divided into tertiles, with cutoff values for the tertiles of 7.61 ng/ml and 24.21 ng/ml. One-way analysis of variance and the chi-square test were used to compare differences among these tertile groups. Logistic regression analysis was used to ascertain associations with PDIA4, with MetS as the dependent variable. In logistic regression analysis, we included potential confounding variables including age, sex, lifestyle variables (history of smoking and alcohol consumption), serum creatinine, uric acid, hsCRP and alanine aminotransferase. Multivariate logistic regression models were used to estimate the odds ratios (ORs) and 95% confidence intervals (CIs) for MetS and the individual components. To validate the diagnostic precision of PDIA4 and other MetS components in prediction of MetS, receiver operating characteristic (ROC) curves were plotted. The area under the curve (AUC) and 95% CI were then calculated for each curve.

## Results

The characteristics of the participants are shown in Table 1. The participants were classified into groups of those with (n = 343) and without (n = 326) MetS. The mean ages of the two groups were 47.82±14.95 and 46.94±15.03 years, respectively. There were significant differences in all anthropometric and basic biochemical values between the two study groups except for age, sex, HOMA-2β and creatinine levels. Compared to those without MetS, those with MetS had a significantly higher serum level of PDIA4 (16.05±8.45 vs. 41.82±20.32 ng/ml, P<0.001).

**Table 1:** Anthropometric and biochemical data of the participants.

	MetS(-)	MetS(+)	p value
	n = 326	n = 343	
Male/Female	191/135	208/135	ns
Age (years)	46.94 (15.03)	47.82 (14.95)	ns
BMI (kg/m^2^)	23.12 (3.38)	27.05 (3.43)	<0.001
Waist circumference (cm)	81.16 (9.57)	91.44 (8.08)	<0.001
Systolic blood pressure (mmHg)	123.05 (15.71)	136.32 (16.88)	<0.001
Diastolic blood pressure (mmHg)	77.33 (9.74)	85.62 (10.13)	<0.001
Glucose 0' (mg/dl)	100.95 (36.08)	132.83 (51.49)	<0.001
Glucose 120' (mg/dl)	156.79 (81.84)	241.1 (95.44)	<0.001
Insulin0' (μIU/Ml)	11.16 (5.35)	18.78 (10.94)	<0.001
Insulin120' (μIU/ml)	74.47 (33.38)	103.17 (51.91)	<0.001
HOHA-2B	153.61 (76.28)	134.71 (50.38)	ns
HOMA-2IR	2.80 (1.58)	6.15 (3.67)	<0.001
HbA1C (%)	5.95 (1.29)	6.85 (1.62)	<0.001
Total cholesterol (mg/dl)	189.43 (34.27)	199.48 (37.9)	0.002
LDL-cholesterol (mg/dl)	122.35 (33.57)	134.32 (33.67)	0.002
HDL-cholesterol (mg/dl)	52.98 (15.25)	39.52 (10.12)	<0.001
Triglyceride (mg/dl)	104.48 (48.8)	207.87 (87.4)	<0.001
Creatinine (mg/dl)	0.81 (0.17)	0.81 (0.19)	ns
Uric acid (mg/dl)	5.09 (1.45)	5.71 (1.52)	<0.001
ALT (U/L)	21.15 (11.95)	32.87 (17.68)	<0.001
hsCRP (ng/ml)	1.28 (0.66)	2.29 (1.36)	<0.001
PDIA4 (ng/ml)	16.05 (8.45)	41.82 (20.32)	<0.001

Data are expressed as mean±SD

**Fig 1:**
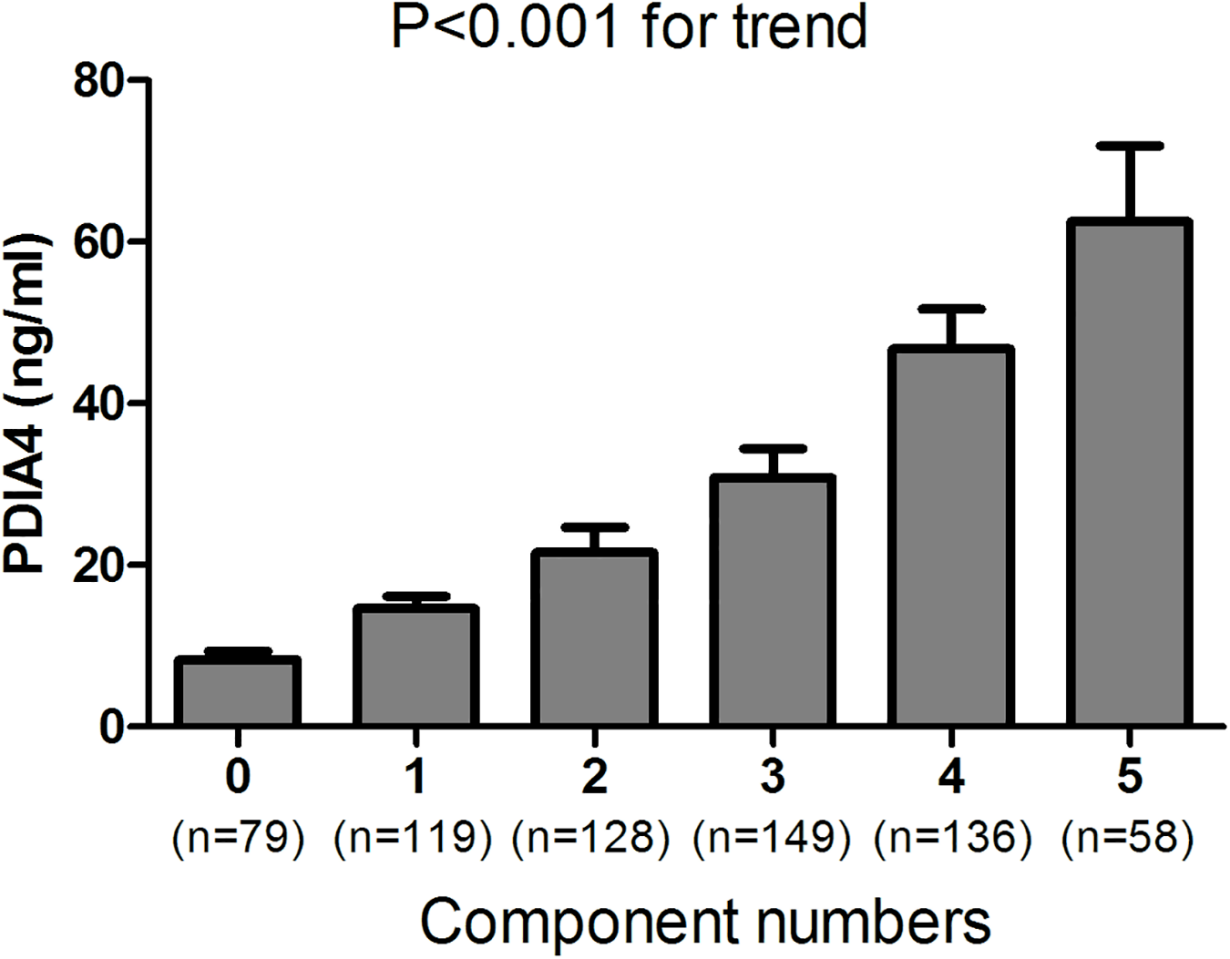
Association of serum PDIA4 levels with the number of MetS components in the study subjects (P<0.001 for trend). Components used to classify individuals with MetS included waist circumference, TG, HDL, blood pressure and fasting blood glucose.

The subjects were further divided into tertiles of PDIA4 level to examine the association between PDIA4 level and MetS. In multivariate logistic regression analysis adjusted for age and sex, serum PDIA4 levels in the 2nd and 3rd tertiles (compared with the bottom tertile) were significantly associated with a higher odds of having MetS (OR 2.41 (95% CI 1.62–3.57) and 9.16 (95% CI 5.94–14.13), respectively) (Table 2). This association remained significant after additional adjustments for lifestyle variables (history of smoking and alcohol consumption), serum creatinine, uric acid, hsCRP and alanine aminotransferase (Table 2). After adjusting for these variables, the adjusted OR and 95% CI for MetS for the individuals with the highest PDIA4 tertile was still 4.83 (95% CI 2.71–8.60) compared with the lowest tertile (Table 2). In addition, the PDIA4 level increased with the number of MetS components (Fig 1). The mean concentrations of PDIA4 in the groups with 0, 1, 2, 3, 4, and 5 MetS components were 8.32, 14.64, 21.58, 30.86, 46.74, and 62.48 ng/ml, respectively (P<0.001 for trend).

**Table 2:** Association of serum PDIA4 with the presence of metabolic syndrome in logistic regression analysis.

	Serum PDIA4, Tertile^a^				
	T1	T2		T3	
		OR	95% CI	OR	95% CI
Model I	Ref	2.42	1.63~3.59	9.25	5.99~14.29
Model II	Ref	2.41	1.62~3.57	9.16	5.94~14.13
Model III	Ref	2.17	1.36~3.46	6.19	3.69~10.40
Model IV	Ref	2.07	1.29~3.31	4.83	2.71~8.60

^a^ Serum PDIA4 tertiles according to cutoff values of 7.61 ng/ml and 24.21 ng/ml

Model I: adjusted for age and sex

Model II: adjusted for Model I and lifestyle factors (smoking and drinking habits)

Model III: adjusted for Model II, creatinine, uric acid, and hsCRP

Model IV: adjusted for Model III and liver function marker (ALT)

Multivariate analysis showed a significantly graded relationship between PDIA4 concentration and individual components of MetS (Table 3). Individuals with the intermediate PDIA4 tertile (T2) had a higher risk of a larger waist circumference (OR = 2.08, 95% CI 1.29–3.35), blood pressure (OR = 1.75, 95% CI 1.12–2.74) and serum TG (OR = 2.63, 95% CI 1.56–4.44) than those with the lowest PDIA4 tertile. Components including waist circumference (OR = 2.41, 95% CI 1.34–4.32), blood pressure (OR = 2.71, 95% CI 1.57–4.67), fasting glucose concentration (OR = 3.17, 95% CI 1.80–5.57) and serum TG (OR = 4.12, 95% CI 2.30–7.37) were most affected by a sharp rise in PDIA4 level (Table 3).

**Table 3:** Adjusted odds ratios and 95% confidence intervals for the individual components of metabolic syndrome by serum PDIA4 level.

	Serum PDIA4, Tertile^a^				
	T1	T2		T3	
		OR	95% CI	OR	95% CI
Waist circumference (men: ≥ 90 cm, women: ≥80 cm)	Ref	2.08	1.29~3.35	2.41	1.34~4.32
Blood pressure ≥130/85 mmHg	Ref	1.75	1.12~2.74	2.71	1.57~4.67
Fasting serum glucose ≥100 mg/dl	Ref	1.10	0.70~1.73	3.17	1.80~5.57
HDL-C (men: <40 mg/dl, women: <50 mg/dl)	Ref	0.98	0.62~1.54	1.49	0.88~2.52
Triglycerides ≥150 mg/dl	Ref	2.63	1.56~4.44	4.12	2.30~7.37

^a^Serum PDIA4 tertiles according to cutoff values of 7.61 ng/ml and 24.21 ng/ml

Adjusted for age, sex, lifestyle factors (smoking and drinking habits), creatinine, uric acid, hsCRP, and liver function marker (ALT)

Compared to the other laboratory criteria for MetS, PDIA4 at a cutoff point of 15.24 ng/ml had relatively high sensitivity and specificity (Table 4). At cutoff point of 15.24 ng/ml, the diagnostic sensitivity and specificity of PDIA4 for the metabolic syndrome were 67 and 72%, respectively, in male patients and 60 and 78%, respectively, in female patients. The AUCs for PDIA4, waist circumference, blood pressure, fasting glucose concentration, serum HDL cholesterol and serum TG are shown in Table 5. The AUC of PDIA4 was similar to the other MetS components, and better than blood pressure.

**Table 4:** Sensitivity and specificity of PDIA4 and metabolic syndrome components for the detection of metabolic syndrome.

Parameter	Sensitivity (%)		Specificity (%)	
	Male	Female	Male	Female
PDIA4≧15.24 (ng/ml)	67	60	72	78
Waist circumference (cm)	72	80	90	68
Blood pressure (mmHg)	77	63	76	76
Fasting glucose (mg/dl)	87	67	74	77
HDL-cholesterol (mg/dl)	74	82	76	74
Triglycerides (mg/dl)	68	89	50	96

**Table 5:** ROC analysis of parameters for metabolic syndrome.

Test result variables	AUC	s.e.	95% CI	
			Lower bound	Upper bound
PDIA4 (ng/ml)	.737	.019	.699	.774
Waist circumference (cm)	.807	.017	.774	.840
Systolic blood pressure (mmHg)	.728	.020	.690	.766
Diastolic blood pressure (mmHg)	.730	.019	.692	.768
Fasting glucose (mg/dl)	.797	.018	.762	.832
HDL-cholesterol (mg/dl)	.782	.018	.748	.816
Triglycerides (mg/dl)	.829	.016	.798	.861

Abbreviations: AUC: area under the curve; CI: confidence interval; HDL: high-density lipoprotein; ROC: receiver operating characteristic.

## Discussion

**A** recent study reported that unresolved ER stress may be involved in a variety of metabolic disorders, including insulin resistance, obesity and type 2 diabetes mellitus.[15] During ER stress, a variety of molecules are upregulated in response to environmental cues including PDIA4. PDIA4 is a member of the PDI family, and dysfunction of the PDI family has been reported to be related to the pathogenesis of numerous diseases in cell and animal models.[19] However, direct clinical evidence of the function of PDIA4 is lacking. This study is the first to report an association between serum PDIA4 concentration and the risk of MetS in human Chinese adults, with a high PDIA4 concentration being strongly associated with MetS. This association was independent of age, gender, inflammation, lifestyle factors, uric acid, renal and liver function.

A growing body of evidences suggest that ER stress and associated molecules are critically involved in lipogenesis and cholesterol metabolism.[28] The function of ER includes protein translation, TG droplet synthesis, and cholesterol and nutrient sensing. Three fatty acids and one glycerol molecule join together to form TG by enzymes resident in the ER membrane, and TG molecules (along with cholesterol) are stored in droplet form. Furthermore, several studies isolating lipid droplets from cells have shown the presence of the ER chaperone protein GRP78 in these droplets,[29, 30] supporting its ER origin. ER stress also induces hepatic steatosis via an increased expression of hepatic very low density lipoproteins (VLDL) receptor (VLDLR) through direct binding of the ATF4 transcription factor to the VLDLR promoter region, leading to the accumulation of TG.[31] In an old mice model, TG were found to be markedly accumulated and lipogenic genes deregulated by ER stress-mediated downregulation of farnesoid X receptor (FXR),[32] which plays a pivotal role in the regulation of hepatic TG metabolism.[33] Tauroursodeoxycholic acid and 4-phenylbutyrate treatment in the old mice reduced the expressions of ER stress markers, but increased FXR expression with reduced hepatic TG content, suggesting that FXR has a protective role in ER stress-mediated lipotoxicity. A previous report [34] demonstrated that ER-resident molecular chaperones interact with apolipoprotein B-100 (apoB) during maturation. The correct folding of apoB into its mature form is a complex process that leads to the formation and secretion of triacylglycerol-rich lipoproteins, chylomicrons, and the atherogenic lipoproteins, VLDL.[35, 36] ApoB has also been shown to exit the ER in complexes containing the chaperones GRP94, ERp72, calreticulin, GRP78, and CyPB, assisting in its early folding stages. Some clues with regards the function of PDIs may be present in these complexes and the direct interaction with apoB.[37] Our results showed a strong relationship between PDIA4 and TG concentrations, and the individuals with the intermediate PDIA4 tertile (T2) had a 2.63-fold higher risk of developing a high TG level, and this risk increased to 4.12-fold for those with the highest tertile of PDIA4 (T3). This study is the first human study to show the clinical relevance of serum PDIA4 with TG level, and reinforces the evidence of preclinical studies that ER stress is involved in TG synthesis and regulation. However, the association between PDIA4 and HDL was not more significant than that between PDIA4 and TG in this study. Recent studies have reported that HDL is a potential inhibitor of ER stress-mediated cell death, especially in pancreatic cells. HDLs have also been shown to prevent palmitate-induced UPR induction, ER stress and cell death through restoration of ER functionality in terms of protein folding and trafficking.[38] HDLs may be able to restore ER function after Ca^2+^ depletion by thapsigargin or palmitate, whereas they may not be able to prevent ER stress induced by the accumulation of misfolded proteins, such as non-glycosylated proteins, after tunicamycin treatment.[39] Taken together, the causal relationship between HDL and ER stress still remains to be confirmed. However, these *in vitro* studies may partially explain our results with regards the correlation between ER stress protein, PDIA4, and HDL in humans.

The etiology or cause of obesity is an imbalance between food intake and energy expenditure, which leads to an excessive accumulation of adipose tissue. Other factors involved in the process of developing obesity include hypoxia, inflammation, free fatty acids, oxidative stress and ER stress. Accumulating evidence indicates that obesity may be highly related to ER dysfunction.[28] Recent studies have reported the upregulation of ER stress markers such as the glucose-regulated protein GRP78, calreticulin, calnexin, and PDI in adipose tissue, the liver, and hypothalamus of obese mice, indicating ER stress in these tissues.[40] In addition, results from *in vitro* and *in vivo* studies have revealed that nutrient excess, a cause of obesity, also triggers ER stress.[15] Nakatani et al. reported that excessive nutrient intake triggered ER stress in the adipose tissue of *ob/ob* mice and mice fed with a high-fat diet.[41] The reason for an increase in ER stress in response to excess nutrient intake may be due to the increased demand for protein and lipid production and structural changes in tissues primarily participating in energy storage, such as adipocytes.[42] It is known that PDI expression in adipose tissue is upregulated in obese individuals compared to lean individuals.[43] It has also been reported that members of the PDI family form a covalent bond with adiponectin, which mediates ER retention.[44] This effect subsequently influences adiponectin secretion and leads to metabolic dysfunction. This suggests that the PDI family may also be involved in metabolic regulation. Our unpublished *in vitro* data showed that the expressions of ER stress markers and PDIA4 were increased during adipogenesis. In response to palmitate treatment, the expression of ER stress markers and PDIA4 were further elevated in murine 3T3-L1 mature adipocytes. Incubating 3T3-L1 pre-adipocytes with PDI inhibitors blocked adipogenesis and palmitate-induced lipid accumulation and inflammation. In this study, we found that the serum PDIA4 concentration was positively correlated with BMI and waist circumference. Furthermore, the individuals with a higher PDIA4 tertiles had more than twice the risk of developing an increased waist circumference. To the best of our knowledge, this is the first study to report an association between a PDI chaperone and obesity.

Insulin resistance is a major characteristic of obesity and type 2 diabetes mellitus, and ER stress appears to directly inhibit insulin signaling pathways. ER stress triggers UPR via three downstream proteins: protein kinase R-like eukaryotic initiation factor 2 kinase (PERK), activating transcription factor 6 (ATF6), and inositol requiring 1> (IRE1α). IRE1α becomes active when phosphorylated in response to ER stress [45] and in turn activates c-Jun terminal kinase (JNK) and IκB kinase, both of which impair insulin signaling by phosphorylating IRS1 on serine residues.[46] PERK, which is activated by saturated acids and lipopolysaccharides, inhibits insulin signaling by directly phosphorylating IRS1 or activating JNK.[47] Nothing is known about the role of PDIA4 in cell growth and viability in pancreatic β-cells, or about its role in diabetes. The expression of PDIA4 has been shown to be increased in β-cells in response to metabolic stress, and to be elevated in the blood of diabetic animals. Moreover, PDIA4 was found to inhibit the development of diabetes in a mouse model.[48] Our results revealed that individuals with a high PDIA4 tertile (T3) had a 3.17-fold higher risk of developing a high fasting glucose level. We also found that hyperglycemia and palmitate increased insulin resistance and induced UPR and PDIA4 signaling in murine C2C12 skeletal muscle cells *in vitro*, and that PDIA4 knockdown prevented the increase in insulin resistance and the expression of glucose transporter 4 (GLUT4) (Lee, et al. unpublished data).

In this study, we found that PDIA4 level, this new ER stress protein, may contribute to the development of obesity-mediated adverse effects that result in clustering of insulin resistance, glucose intolerance, dyslipidemia and hypertension. The main limitation of this study is the cross-sectional design, and longitudinal studies are required to validate the diagnostic usefulness of PDIA4 and other ER stress-related proteins for the detection of subjects at risk of developing MetS. Furthermore, as the expression of MetS may be dependent on several factors that are linked to ethnicity, it would also be necessary to establish cutoff levels of PDIA4 for the diagnosis of MetS in different populations.

In conclusion, we found that serum PDIA4 levels were associated with an increased risk of MetS in Chinese adults. In view of the current controversy with regards the precise definition, diagnostic criteria and pathophysiology of MetS,[49] some physicians may take issue with including another criterion for the definition of MetS. In this study, we demonstrated that PDIA4 is associated with obesity, insulin resistance and dyslipidemia, and that it was positively associated with the expression of MetS in our study population. Furthermore, our results suggest that PDIA4 may be a novel therapeutic target for diseases associated with MetS. Therefore, future studies should focus on ways to modulate PDIA4 activity to reach a desired therapeutic benefit.
